# The Physician and the State

**Published:** 1884-02-20

**Authors:** 


### *THE PHYSICIAN AND THE STATE.*

The forcible extraction of the opinions of medical men as experts on the witness-stand, without compensating them for such opinions, is a matter that ought to meet with the opposition and protest of the medical profession everywhere. The knowledge of the physician thus obtained, is, in one sense, his private property, for which he has incurred, it may be, large expense with years of study and labor. Can it be rightly taken from him without compensation, even though it be in the furtherance of law and of justice. We opine not.

Dr. Garrison, in a paper upon this subject (Medical Bulletin) thus states the relations between medical men and the law:

1. "As a Plaintiff. There is nothing in the profession peculiar to the physician. A 'visit' per se, is not a valuable consideration, and therefore not a lawful demand. 'A professional visit, at the request of defendant,' is recommended as a proper form.

"The Defense: He did not cure or benefit the defendant, is no bar to recovery, as skill and care, not cure or benefit, are the conditions of the implied contract.

2. "As a Defendant: The law presumes that a physician agrees to furnish the fair average skill of the craft, not the highest known to the profession.

3. "As a Witness: This may be ordinary or expert. The ordinary witness testifies only in regard to what he saw, heard, or observed in the case, the same as any other witness. As expert, the position of the physician is judicial, and he should be called by the court, and not by the contestants."

